# Clinical and molecular characteristics, risk factors and outcomes of Carbapenem-resistant *Klebsiella pneumoniae* bloodstream infections in the intensive care unit

**DOI:** 10.1186/s13756-017-0256-2

**Published:** 2017-10-02

**Authors:** Xia Zheng, Jian-feng Wang, Wang-lan Xu, Jun Xu, Juan Hu

**Affiliations:** 1 Intensive Care Unit, The First Affiliated Hospital, College of Medicine, Zhejiang University, 79 Qingchun Road, Hangzhou, 310003 People’s Republic of China; 2 Department of Respiratory Diseases, The Affiliated Hospital of Hangzhou Normal University, Hangzhou, Zhejiang, No. 126 Wenzhou Road, Hangzhou, 310009 People’s Republic of China; 3 Intensive Care Unit, Hospital of Zhejiang General Corps of Armed Police Forces, No. 16 Nanhu Road, Jiaxing, 314000 Zhejiang, People’s Republic of China

## Abstract

**Background:**

To analyze the clinical characteristics and outcomes of carbapenem-resistant *Klebsiella pneumoniae* (CRKp) and carbapenem-susceptible *K. pneumoniae* (CSKp) bloodstream infections (BSIs), and to study the risk factors for development of CRKp BSI and *K. pneumoniae* BSI-related mortality.

**Methods:**

A retrospective case control study of patients with *K. pneumoniae* BSI was conducted in the intensive care unit of the First Affiliated Hospital, Medical of College, Zhejiang University from January 2013 to December 2014. Carbapenem resistance was defined in accordance with the Clinical and Laboratory Standards Institute 2016 guidelines. Risk factors for the development of CRKp BSI and risk factors for mortality due to *K. pneumoniae* BSI were assessed. Virulence genes were detected by polymerase chain reaction assay.

**Results:**

In total, 48 patients were enrolled in the study, including 31 (65%) patients with CRKp BSI and 17 (35%) patients with CSKp BSI. CSKp infection was associated with more severe clinical symptoms, particularly a higher serum creatinine level (165.06 ± 127.01 in the CSKp group vs. 93.77 ± 84.35 μmol/L in the CRKp group, *p =* 0.039), but there was no significant difference in prognosis between the CSKp and CRKp groups. On multivariate analysis, indwelling central venous catheter (*p =* 0.045) was the only factor independently associated with CRKp bacteremia. However, the mortality of *K. pneumoniae* BSI patients was not correlated with carbapenem resistance. In addition, the isolates had diverse clonality and different origins. The frequency of detection of the *allS* and *magA* virulence genes was higher in the CSKp group than in the CRKp group (*alls p =* 0.04; *magA p =* 0.047).

**Conclusions:**

Patients in the CSKp group experienced more severe clinical symptoms, although mortality did not differ significantly between the CRKp and CSKp groups. An indwelling central venous catheter was the only factor independently associated with CRKp BSI. The mortality of patients with *K. pneumoniae* BSI was not associated with carbapenem resistance. The frequency of virulence genes was higher in the CSKp group than in the CRKp group.

## Background

*Klebsiella pneumoniae*, a member of the family Enterobacteriaceae, is a causative organism of various infections, including serious community-onset infections, such as necrotizing pneumonia, pyogenic liver abscesses, and endogenous endophthalmitis [1, 2]; and nosocomial infections, particularly urinary tract infections (UTIs), respiratory tract infections, and bloodstream infections (BSIs) [1, 3, 4]. Due to abuse of antimicrobial agents in developing countries, the incidence of carbapenem-resistant Enterobacteriaceae (CRE) is of considerable concern. *K. pneumoniae* is the most prevalent Enterobacteriaceae species, accounting for 71.9% of 242 CRE strains in a retrospective study conducted in a tertiary hospital in Hangzhou, China [5]. Surveillance of antibiotic resistance by the China CHINET showed that 2.9% and 2.8% of *Klebsiella s*pp. were resistant to imipenem and meropenem, respectively, in 2005 compared to 10.5% and 13.4%, respectively, in 2014 [6]. The production of carbapenemases (e.g., KPC, NDM, VIM, OXA-48-like) is the most common mechanism of resistance among *K. pneumoniae* isolates. Other mechanisms include alterations in outer membrane permeability, mediated by loss of porins and the upregulation of efflux systems [7].

The mortality rate of carbapenem-resistant *Klebsiella pneumoniae* (CRKp) infections in North America, South America, Europe, and Asia is reportedly 33.24%, 46.71%, 50.06%, and 44.82%, respectively [8]. Similarly, a study done in Shanghai, China showed that the 28-day mortality and in-hospital mortality rates of CRKp BSI patients were significantly higher than those of patients with carbapenem-susceptible *K. pneumoniae* (CSKp) BSIs (33.3% vs. 16%, *p =* 0.04; 42.4% vs. 24.6%, *p =* 0.005, respectively) [9]. Although CRKp is reportedly associated with prolonged hospitalization and mortality [8, 10–12] because such patients typically receive inappropriate empiric therapy, other studies found no such relationship [13–15].

There are various risk factors for CRKp BSI. A study conducted in a teaching hospital in Shanghai, China suggested that skin and soft tissue infection (odds ratio [OR] 26.63 and ICU-acquired infection (OR 5.82) was a risk factor for CRKp BSI [9]. multisite colonization (hazard ratio [HR] 13.73), ICU stay (HR 3.14) and previous BSI (HR 6.62) was associated with the development of CRKp BSI in colonized patients [16]. Primary liver disease and hepatitis C virus infection or hepatocellular cancer were significantly associated with development of CRKp in intensive care unit (ICU) patients after orthotopic liver transplantation [17]. Even no exposure independently predicted CRKp BSI in carriers of CRKp [18].

Similarly, several factors are reportedly associated with mortality related to *K. pneumoniae* BSI. The lung as the probable source of infection (OR 4.23) and a high Sequential Organ Failure Assessment (SOFA) score (OR 1.40) were strong prognostic factors for crude 28-day *K. pneumoniae* BSI mortality in a teaching hospital in Shanghai, China [9]. Septic shock (HR 3.86), acute respiratory failure (HR 2.32), inadequate initial antimicrobial therapy (HR 1.87) and carbapenem resistance by *K. pneumoniae* isolates (HR 1.85) were independently associated with mortality in onco-hematological patients [19]. In a univariate analysis, Acute Physiology and Chronic Health Evaluation (APACHE II) score, SOFA score, and CRKp BSI were predictive of ICU mortality after orthotopic liver transplantation [17].

It is generally accepted that CRKp BSIs are associated with high mortality, mostly because of the paucity of antimicrobials active against CRKp and the multiple comorbidities of patients [20]. Severe infection causes organ dysfunction and/or failure via complex mechanisms, including pathogenic microorganisms, an excessive inflammatory response, and immune dysfunction. However, antimicrobial resistance does not always lead to organ dysfunction and/or failure [21]. The immune system plays an important role in disease manifestations, with multiple contributing factors, some of which may not be accounted for by routinely collected data. At present, whether the systemic manifestations of infection and frequency of bacterial virulence genes differ between CRKp and CSKp BSI patients is unclear. Therefore, the objective of our study was to compare the prognosis and clinical characteristics of patients with CRKp and CSKp infections in the ICU, identify risk factors for the development of CRKp BSI and mortality of *K. pneumoniae* BSI, and assess the frequency of bacterial virulence genes in patients with CRKp and CSKp BSI.

## Methods

### Study design and patients

This retrospective case-control study was conducted at the First Affiliated Hospital of the Medical College, Zhejiang University, a 2500-bed tertiary-care teaching hospital, and included all adult patients with BSI caused by *K. pneumoniae* and hospitalized in the 30-bed medical ICU from January 1, 2013 to December 31, 2014.

The patients were identified using the records of the clinical microbiology laboratory. All patients with a positive blood culture for *K. pneumoniae* was included in the study. Infective symptoms and signs were compatible with systemic inflammatory response syndrome (SIRS; i.e., fever or hypothermia, respiratory rate > 20 breaths per minute, tachycardia >90 beats/min, and white blood cell count >11,000 ml or <4000/ml, using the 1999 criteria). If more than one episode occurred in the same patient, only the first episode was included in the study [22].

Cases with incomplete medical records were excluded from the study. From 2013, the identification and antimicrobial susceptibility testing of all blood *K. pneumoniae* isolates were performed using Vitek 2 panels (bioMerieux, France); isolates were stored at −80 °C [23]. Reserved strains were retrospectively tested for the presence of specific virulence genes.

The patients were divided into the CRKp and CSKp groups. Carbapenem resistance was defined as a minimum inhibitory concentration of ≥4 mg/L for meropenem or imipenem or ≥2 mg/L for ertapenem; other strains were defined as carbapenem-susceptible. Data interpretation was performed in accordance with the Clinical and Laboratory Standards Institute (CLSI) 2016 guidelines.

To identify risk factors for the development of CRKp BSI and m related ortality, the following data were recorded: demographics (sex, age), comorbidities, history of surgery, hospital or ICU admission in the last 30 days, use of steroids or immune modulators, antibiotic exposure history, and indwelling prosthetic material. In addition, for assessment of severe infection, APACHE II scores, liver function, kidney function, and inflammatory markers, at admission and at the time of positive blood culture, were recorded [17, 19].

The primary outcomes were crude survival rates at 7, 14, and 28 days. Secondary outcomes were current ICU stay duration, bacterial clearance rate, and duration of mechanical ventilation [24].

### Multilocus sequence typing and pulsed-field gel electrophoresis

According to the multilocus sequence typing (MLST) scheme of *K. pneumonia*, seven conserved housekeeping genes (*gapA*, *infB*, *mdh*, *pgi*, *phoE*, *rpoB*, and *tonB*) were amplified and sequenced [25]. Pulsed-field gel electrophoresis (PFGE) was performed using *Xba*I (Dalian Takara Bio Inc., China), as described previously. To identify isolates associated with outbreaks, PFGE band patterns were interpreted according to the criteria proposed by Tenover et al. [26, 27].

### Detection of virulence genes by polymerase chain reaction

The K1, K2, K5, K20, K54, and K57 capsular serotypes were detected by polymerase chain reaction (PCR), as described previously. Virulence genes (*magA*, *rmpA*, *rmpA2*, and *allS*) were detected by PCR using primers, as described previously. PCR products were interpreted and sequenced [23].

### Statistical analysis

Statistical analyses were performed using SPSS software (ver. 18.0; SPSS Inc., USA). Continuous variables are presented as means ± SD and were evaluated by Student’s *t*-test or the Mann-Whitney U test, as appropriate. A chi-squared test or Fisher’s exact test was used for categorical variables, and multivariate analyses were performed using logistic regression models to identify independent risk factors for the outcome variables. All biologically plausible variables significant at *p* < 0.10 in univariate analysis were entered into a multivariate forward logistic regression analysis. A *p* value <0.05 was considered to indicate statistical significance.

## Results

### Demographics of the study population

Between January 1, 2013 and December 31, 2014, 48 patients had at least one episode of *K. pneumoniae* BSI in the ICU of our hospital. Thirty-three bloodstream isolates were included in this study; the others were excluded due to incomplete clinical information. The mean age in the CRKp and CSKp groups was 57.61 ± 14.78 and 62.71 ± 16.34 years, respectively (*p =* 0.306). Male patients accounted for 79% of the patients (56% in the CRKp group and 23% in the CSKp group, *p =* 0.502). To assess the frequency of bacterial virulence genes, 33 isolates (21 resistant and 12 susceptible) were obtained from 33 patients (17 and 4 male patients from the CRKp and CSKp groups, respectively [*p =* 0.357]). The mean age of these patients was 58.14 ± 15.16 and 65.58 ± 15.57 years, respectively (*p =* 0.190).

### Clinical symptoms and prognostic factors of *K. pneumoniae* BSI

APACHEII score, peripheral blood leukocyte count, and C-reactive protein level were higher in the CSKp group than in the CRKp group at the time of bacteremia, albeit not significantly so. Many indexes showed the deteriorative tendencies in CSKp, including coagulation function, liver and kidney function, however only the static difference occurred in serum creatinine level (165.06 ± 127.01 in the CSKp group vs. 93.77 ± 84.35 μmol/L in the CRKp group, *p =* 0.039) (Table 1).

**Table 1:** Clinical symptoms of *K. pneumoniae* BSI

Variable	CSKp (*n* = 17)	CRKp (*n* = 31)	*P*
APACHEII score(mean ± SD)	18.18 ± 7.48	15.03 ± 6.38	0.089
WBC 10E9/L (mean ± SD)	14.24 ± 13.63	13.99 ± 9.66	0.497
leukocytes count (mean ± SD)	81.20 ± 14.60	88.02 ± 7.95	0.180
CRP(mg/l)(mean ± SD)	162.49 ± 118.07	113.74 ± 79.63	0.191
ALT (mean ± SD)	152.88 ± 399.23	49.84 ± 50.05	0.359
AST (mean ± SD)	490.82 ± 1655.64	49.63 ± 48.54	0.088
Total bilirubin (mean ± SD)	73.06 ± 142.17	62.68 ± 128.66	0.754
Direct bilirubin (mean ± SD)	43.29 ± 83.58	39.48 ± 86.63	0.779
PT (s) (mean ± SD)	17.71 ± 10.54	15.05 ± 5.27	0.439
Creatinine (mean ± SD)	165.06 ± 127.01	93.77 ± 84.35	0.039
Urea nitrogen (mean ± SD)	14.42 ± 9.88	14.40 ± 10.05	0.931

*Abbreviations*: *CSKp* carbapenem-susceptible *Klebsiella pneumoniae*, *CRKp* carbapenem-resistant *Klebsiella pneumoniae, WBC* white blood cell, *CRP* C-reactive protein, *ALT* alanine transaminase, *AST* aspartate transaminase, *PT* prothrombin time

The prognostic factors of the CRKp and CSKp BSI patients are presented in Table 2. The duration of mechanical ventilation of the CRKp and CSKp groups was 18.50 ± 31.91 and 28.72 ± 31.06 days, respectively (*p =* 0.127). The current ICU stay duration was similar between the CRKp and CSKp groups (21.47 ± 33.67 vs. 31.74 ± 30.75 days, respectively [*p =* 0.073]). The bacterial clearance rate was 32% and 35% in the CRKp and CSKp groups, respectively (*p =* 0.718). The survival rate of the CRKp group was 74% at 7 days, 68% at 14 days, and 61% at 28 days, compared to 65% at 7 days, 59% at 14 days, and 47% at 28 days in the CSKp group (*p =* 0.489, 0.537, and 0.342, respectively).

**Table 2:** Clinical outcomes of *K. pneumoniae* BSI

Variable	CSKp (n = 17)	CRKp (*n* = 31)	*P*
Duration of mechanical ventilation (mean ± SD)(d)	18.50 ± 31.91	28.72 ± 31.06	0.127
Current ICU stay	21.47 ± 33.67	31.74 ± 30.75	0.073
Bacterial clearance rate	6(35%)	10(32%)	0.718
7-day survival rate	11(65%)	23(74%)	0.489
14-day survival rate	10(59%)	21(68%)	0.537
28-day survival rate	8(47%)	19(61%)	0.342

*Abbreviations*: *CSKp* carbapenem-susceptible *Klebsiella pneumoniae*, *CRKp* carbapenem-resistant *Klebsiella pneumoniae, ICU* intensive care unit

### Risk factors for the development of CRKp BSI

In univariate analyses, development of CRKp BSI was significantly associated with central venous catheterization and an indwelling urinary catheter, but not with comorbidities (including diabetes mellitus, hypertension, coronary heart disease,chromic liver disease, chronic renal failure, solid organ tumor, history of surgery, prior healthcare-associated exposure, exposure to glucocorticoids and/or immunosuppressive drugs, trachea cannula or tracheotomy, an indwelling nasogastric tube, a drainage tube at multiple sites, and APACHE II score at admission). Hepatic and renal function at admission showed a trend towards being associated with the development of CRKp BSI. Although the majority of CRKp BSI patients had been exposed to antimicrobials before the positive culture, only exposure to tigecycline, imipenem and meropenem were included in the multivariate analysis of CRKp BSI (Table 3).

**Table 3:** Univariate analysis of risk factors for CRKp BSI

Variable	CSKp (n = 17)	CRKp (n = 31)	Univariate analysis		
			Adjusted OR	95% CI	*P*-value
Sex (male, n)	11(65%)	27(87%)	0.611	0.144—2.596	0.502
Age (y) (mean ± SD)	62.71 ± 16.34	57.61 ± 14.78			0.306
Co- morbidities					
Diabetes mellitus	5 (29%)	9 (29%)	0.982	0.268—3.602	0.978
Hypertension	9 (53%)	15 (48%)	0.833	0.255—2.724	0.763
Coronary heart disease	1 (6%)	3 (10%)	1.714	0.164—17.886	1
Chronic liver disease	3(18%)	6(19%)	1.120	0.242–5.186	1
Chronic renal failure	2 (12%)	4 (13%)	1.111	0.182—6.796	0.909
Solid organ tumor	4 (24%)	7 (23%)	0.948	0.233—3.850	0.940
HIV	0	0	NA	NA	NA
Surgery in the past medical history^a^	3 (18%)	5 (16%)	0.897	0.186—4.322	0.893
Prior healthcare-associated exposure	7 (41%)	9 (29%)	0.584	0.169—2.017	0.393
Prior medicine exposure					
Glucocorticoid	7 (41%)	13 (42%)	1.032	0.311—3.428	0.959
Immunosuppressor^b^	1 (6%)	2 (6%)	1.103	0.093—13.135	1
Prior use of antimicrobials					
Penicillin	3 (18%)	10 (32%)	2.105	0.480—9.237	0.318
Second cephalosporin	1 (6%)	4 (13%)	2.240	0.228—22.051	0.838
extended-spectrum cephalosporins	7 (41%)	15 (48%)	1.224	0.351—4.269	0.750
Aminoglycosides	1 (6%)	3 (10%)	1.615	0.153—17.016	1
Quinolones	1 (6%)	3 (10%)	1.615	0.153—17.016	1
Tigecycline	0	4 (13%)	0.625	0.492—0.795	0.613
Imipenem	1 (6%)	11(39%)	8.556	0.984—74.408	*0.064*
Meropenem	1 (6%)	12 (39%)	9.882	1.141—85.619	*0.068*
Ertapenem	0	0	NA	NA	NA
Linezolid	1 (6%)	12 (39%)	9.882	1.141—85.619	*0.068*
Glycopeptides Vancomycin	1 (6%)	8 (26%)	5.333	0.599—47.468	0.216
Teicoplanin	1 (6%)	5 (16%)	2.917	0.309—27.560	0.333
Invasive procedure before					
Central venous catheterization	10(59%)	30 (97%)	21.000	2.294—192.225	*0.003*
Trachea cannula	4(24%)	10 (32%)	1.548	0.401—5.971	0.525
Tracheotomy	5(29%)	12 (39%)	1.516	0.426—5.393	0.519
Urinary catheter	7(41%)	23 (74%)	4.107	1.168—14.436	*0.024*
stomach tube	14(82%)	27 (87%)	1.446	0.283—7.384	0.656
Intrathoracic drain	0(0)	4 (13%)	0.614	0.485—0.776	0.282
Abdominal cavity drainage-tube	5(29%)	15 (48%)	2.250	0.639—7.923	0.202
External ventricular drainage tube care	0(0)	1 (3%)	0.612	0.490—0.765	1
Liver function on admission	5(35%)	3 (10%)	0.257	0.053—1.252	*0.079*
Renal function on admission	7(41%)	4(13%)	0.275	0.071—1.070	*0.055*
APACHE II score on admission	12.29 ± 7.32	9.45 ± 4.60			0.334

*Abbreviations*: *CSKp* carbapenem-susceptible *Klebsiella pneumoniae*, *CRKp* carbapenem-resistant *Klebsiella pneumoniae*, *OR* odds ratio, *CI* confidence interval, *HIV* human immunodeficiency virus, *APACHE* Acute Physiology and Chronic Health Evaluation

^a^History of surgery and non-invasive procedures, such as endoscopy or colonoscopy, are excluded from this category

^b^Immunosuppressors: Immunosuppressive drugs, immunosuppressive agents or antirejection medications are drugs that inhibit or prevent activity of the immune system, including cytostatics, antibodies, drugs acting on immunophilins, etc. (https://en.wikipedia.org/wiki/Immunosuppressive_drug)

Variables significant at *p* < 0.10 in the univariate analyses were included in the multivariate analysis, and were as follows: an indwelling central venous catheter, indwelling urinary catheter, exposure to linezolid, imipenem and meropenem, and liver and kidney function at admission. In the multivariate analysis, central venous catheterization was the only independent factor for CRKp BSI. Exposure to imipenem and meropenem were likely related to CRKp BSI, but the association was not significant due to the small sample (Table 4).

**Table 4:** Multivariate analysis of risk factors for CRKp BSI

Variable	Adjusted OR	95% CI	*P*-value
Indwelling central venous catheters	14.343	1.063—193.444	0.045
Exposure to meropenem	11.968	0.756—69.720	0.086
Exposure to imipenem	7.258	0.945—151.497	0.055

*Abbreviations*: *OR* odds ratio, *CI* confidence interval

### Risk factors for mortality of *K. pneumoniae* BSI patients

In univariate analyses, the mortality of *K. pneumoniae* BSI patients was significantly associated with the APACHE II score on the day of bacteremia, but not with CRKp. Liver failure on the day of bacteremia and trachea cannula showed a trend towards being associated with a higher mortality rate. Variables significant at *p* < 0.10 in the univariate analyses were included in the multivariate analysis. However, no factor was identified as being associated with the mortality of *K. pneumoniae* BSI patients in the multivariate analysis, likely due to the small sample size (Table 5).

**Table 5:** Univariate analysis of the 28-day mortality rate of *K. pneumoniae* BSI patients in the ICU

Variable	Died (*n* = 21)	Survived (*n* = 27)	Univariate analysis		
			*P*	OR	95% CI
Sex (male, n)					
Age (y) (mean ± SD)	57.74 ± 16.63	61.57 ± 13.69	0.448		
APACHE II score at admission	11.50 ± 5.53	10.07 ± 4.95	0.406		
APACHE II score on the day of bacteremia	18.29 ± 7.36	14.48 ± 6.11	0.031		
Liver failure^a^ at admission	7	8	0.784	1.188	0.348—4.051
Kidney failure^b^ at Admission	4	7	0.574	0.672	0.168—2.694
Liver failure on the day of bacteremia	11	7	0.06	3.143	0.933—10.584
Kidney failure on the day of bacteremia	6	6	0.614	1.400	0.377—5.195
Co-infection	14	20	0.575	0.700	0.2—2.445
CRKp	12(57.1%)	19(70.3%)	0.342	0.561	0.170—1.856
Diabetes mellitus	5(23.8%)	9(23.3%)	0.371	0.556	0.152—2.027
Hypertension	10(47.6%)	14(51.9%)	0.771	0.844	0.270—2.644
Coronary heart disease	2(9.5%)	2(7.4%)	0.792	1.316	0.170—10.208
Chronic renal failure	2(9.5%)	4(14.8%)	0.582	0.605	0.1—3.672
Solid organ tumor	4(19%)	7(25.9%)	0.574	0.672	0.168—2.694
Surgery in the past medical history	2(9.5%)	6(22.2%)	0.242	0.368	0.066—2.050
Prior healthcare-associated exposure	8(38.1%)	8(29.6%)	0.537	1.462	0.437—4.889
Glucocorticoid	8	12	0.658	0.769	0.240—2.460
Immunosuppressor	2(9.5%)	1(3.7%)	0.409	2.737	0.231—32.430
Central venous catheterization	17(81%)	23(85.2%)	0.696	0.739	0.161—3.383
Trachea cannula	9(42.9%)	5(18.5%)	0.066	3.3	0.899—12.108
Tracheotomy	6(27.3%)	11(40.7%)	0.325	0.545	0.162—1.833
Urinary catheter	13(61.9%)	17(16.9%)	0.940	0.956	0.295—3.102
Abdominal cavity drainage- tube	6(28.6%)	14(51.9%)	0.105	0.371	0.111—1.247
Stomach tube	18(85.7%)	23(85.2%)	0.959	1.043	0.305—0.593
Intrathoracic drain	0	4(14.8%)	0.121	0.523	0.394—0.693
External ventricular drainage tube care	1(4.7%)	0	0.438	0.426	0.305—0.593

*Abbreviations*: *OR* odds ratio, *CI* confidence interval, *CRKp* carbapenem-resistant *Klebsiella pneumoniae*

^a^Liver failure was defined as a serum bilirubin level of >3 mg/dl and/or prothrombin time (PT) of <50% on day 5 after surgery or thereafter; in patients with jaundice, it was defined as an increase in the serum bilirubin level or a PT < 50% on day 5 or thereafter [53]

^b^Kidney failure was defined as a creatinine level of ≥2 mg/dl or requirement for dialysis [33]

### Molecular characteristics of CRKp and CSKp BSI

**Fig. 1:**
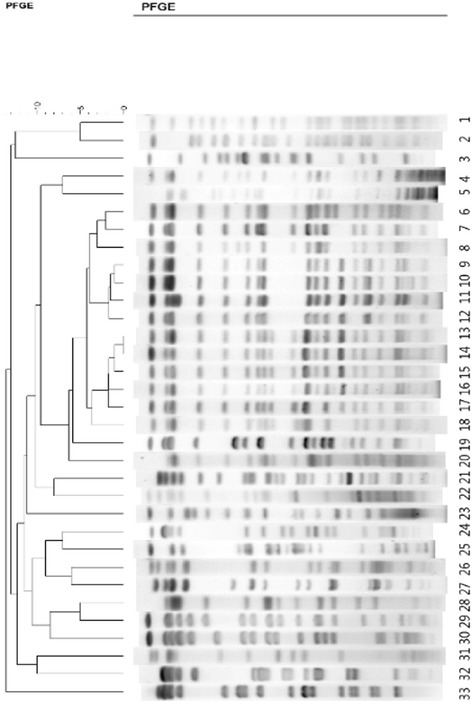
Pulsed-field gel electrophoresis (PFGE) results. Pulsotypes of 33 *K. pneumoniae* blood isolates. The PFGE patterns revealed that the 33 isolates had different origins

**Fig. 2:**
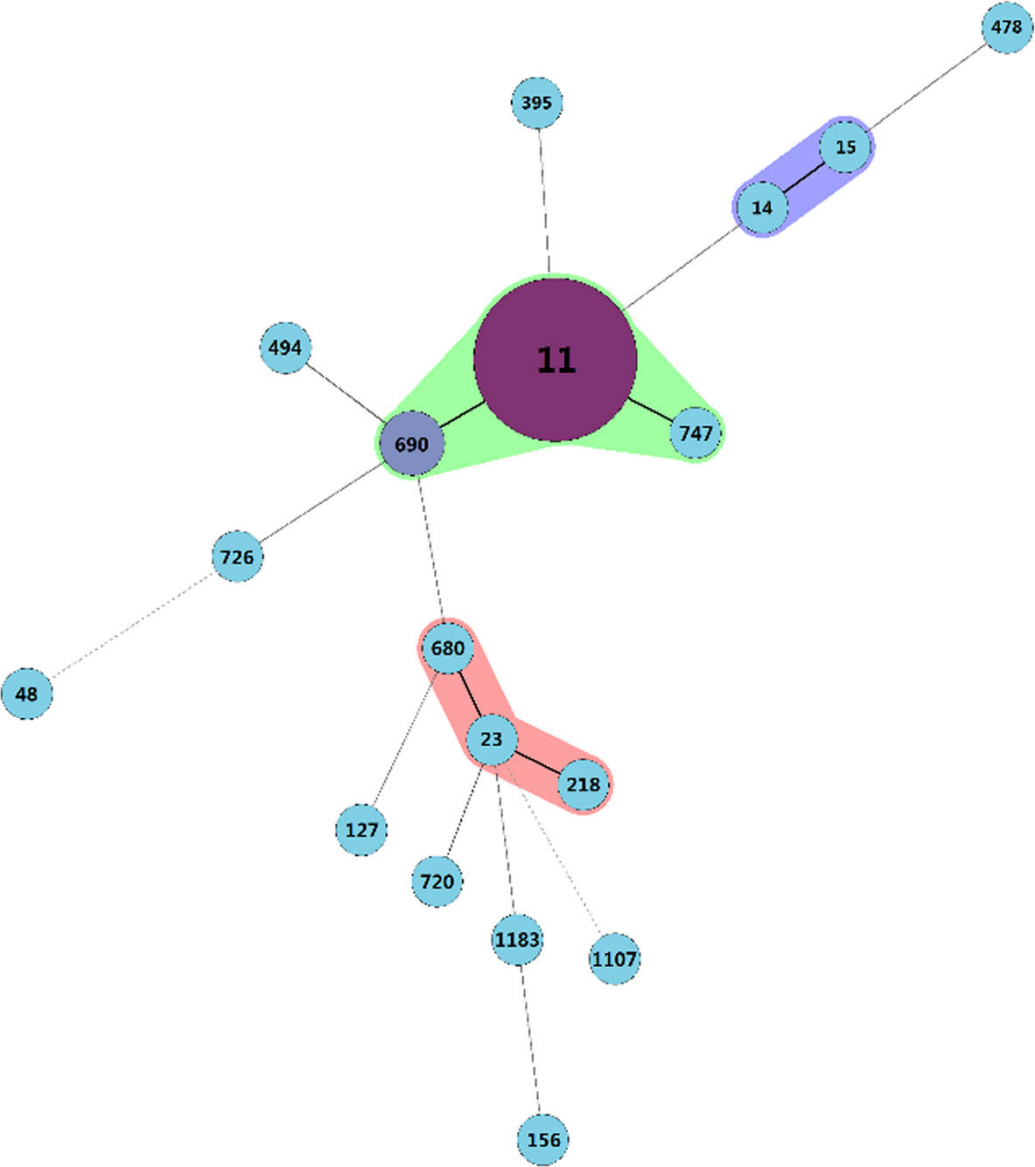
Multilocus sequence typing (MLST) results. Eighteen sequence types (STs) of 33 *K. pneumoniae* blood isolates. MLST indicated considerable clonal diversity; 18 STs were detected, of which ST11 comprised the majority

The PFGE patterns revealed that the 33 isolates had different origins (Fig. 1). MLST revealed considerable clonal diversity; 18 sequence types (STs) were detected (Fig. 2), of which ST11 comprised the majority.

The CSKp isolates harbored the *rmpA2*, *allS*, *K1*, and *magA* virulence genes, while the CRKp isolates possessed *rmpA2*, *magA*, and *K5*, but not *allS* or *K1*. *rmpA*, *K2*, *K20*, *K54*, and *K57* were not detected. The frequency of detection of *allS* and *magA* was higher in the CSKp group than in the CRKp group (*allS p =* 0.04; *magA p =* 0.047) (Table 6).

**Table 6:** Frequency rates of bacterial virulence genes in 33 isolates

Variable	CSKp (*n* = 12)	CRKp (n = 21)	P
rmpA2	3	4	0.686
Alls	3	0	0.04
K1	1	0	0.364
K5	0	1	1
magA	4	1	0.047
K1+ magA	1	0	0.364
rmpA2 + magA	1	0	0.364
alls + magA	1	0	0.364
rmpA	0	0	–
K2	0	0	–
K20	0	0	–
K54	0	0	–
K57	0	0	–

*Abbreviations*: *CSKp* carbapenem-susceptible *Klebsiella pneumoniae*, *CRKp* carbapenem-resistant *Klebsiella pneumoniae*

## Discussion

CRKp BSI is a global public health problem that has been increasing in recent times, and is responsible for considerable morbidity [20, 28, 29]. The incidence of *K. pneumoniae* BSIs in ICU patients exceeds that of *Escherichia coli* BSIs [30, 31]. The mortality rate of critical patients with *K. pneumoniae* BSIs in the ICU was reported to rise up to 67.6% [30].

Population-based screening for *K. pneumoniae* bacteremia was conducted in the Calgary Health Region (population, 1.2 million) from 2000 to 2007. Dialysis, solid-organ transplantation, chronic liver disease, and cancer were risk factors for *K. pneumoniae* bacteremia [10]. CRE surveillance in Michigan healthcare facilities showed that cardiovascular disease, renal failure, and diabetes mellitus were the most frequently reported comorbidities, and risk factors for CRE included surgery within the previous 90 days, recent infection or colonization with a multidrug-resistant organism, and recent exposure to antimicrobials, particularly third- or fourth-generation cephalosporins [32]. The more frequent hospital contact associated with serious comorbidities may result in exposure to, and possibly infection by, nosocomial microorganisms. Moreover, severe chronic comorbidities were more frequent among patients with CRKp BSIs, but chronic comorbidities were not risk factors for CRKp BSI [33]. Similarly, in this study, comorbidities were not independent risk factors for CRKp BSI.

Glucocorticoids and immunosuppressors were not independent risk factors for CRKp BSI; however, previous studies reported different results. In one study, only prior carbapenem administration (*p =* 0.003), was significantly associated with CRKp infection, and another study revealed that the type of antibiotic used before infection—such as third-generation cephalosporins, macrolides and quinolones—was an independent risk factor for CRKp (*p* < 0.05) [34, 35]. Indeed, prior use of macrolides and antibiotic exposure for ≥14 days were the only factors independently associated with nosocomial CRKp bacteremia [33]. In another case-case control study, exposure to quinolones was not associated with CRKp infection, and colonization by CRKp and use of carbapenems were risk factors for infection with CRKp [36]. However, in our study, no antibiotic was a risk factor for CRKp BSI. Different in definitions, the duration of exposure to antibiotics, or different drug-treatment populations among these studies may account for the divergent findings.

In terms of invasive procedures, only an indwelling central venous catheter and urinary catheter were associated with CRKp BSI in univariate analyses. In the multivariate analysis, an indwelling central venous catheter was the only factor independently associated with CRKp BSI, partly consisted with the past studies [37, 38]. However, previous studies indicated that other variables, such as mechanical ventilation and a nasogastric tube, were related to CRKp BSI [38, 39, 40].

Liver and kidney function indices at admission were higher in the CSKp group than in the CRKp group in this study, similar to some previous studies [41, 42]. However, reduced liver and kidney function was not associated with CRKp BSIs.

To improve outcomes, and where there is a need to avoid unnecessary antibiotics so as to reduce CRKp emergence, greater efforts should be made to ensure that initial appropriate antibiotic therapy is delivered to critically ill infected patients, and antibiotic de-escalation should be practiced to avoid unnecessary antibiotic exposure [43]. Moreover, control of the infection source is important for reducing the incidence of BSI. Central line (CL)-associated BSIs in ICUs result in increased morbidity and mortality, and are largely preventable; thus, preventive measures for catheter-related infection are important. Such measures can be applied at central line insertion and maintenance. For example, use of maximal sterile barrier precautions and/or avoiding the femoral vein were applied to reduce the risk of central venous catheter-related bloodstream infection. Moreover, use of central lines should be reduced wherever possible, such as by daily assessment of the need for a CL and timely removal of an unnecessary CL [44].

Infection-related mortality involves a number of factors, including host defense, virulence of the pathogen, source location and control, and the efficacy of available antimicrobials. A recent meta-analysis showed that patients with CRKp had a significantly higher mortality rate than those with CSKp (42.14 vs. 21.12, *p* < 0.001) [45]; this suggests that antimicrobial resistance is related to mortality. However, severe infections are not necessarily caused by drug-resistant bacteria, i.e., antimicrobial resistance is not linked with infection-induced organ dysfunction or failure, or with mortality, in critically ill patients. Therefore, the difference in clinical features and risk factors between CSKp and CRKp BSIs is intriguing.

In our study, the CSKp BSI patients had more severe clinical characteristics, such as higher APACHE II scores and lower alanine transaminase (AST) levels. Indeed, the serum creatinine level at the time of the positive culture was significantly higher in the CSKp group than in the CRKp group. However, CSKp BIS patients had only a trend towards a higher mortality rate. Therefore, although CS-Kp infection can lead to worse clinical symptoms, the mortality rate is similar between the two groups, despite there being fewer therapeutic options for CRKp BSI.

The risk factors for mortality due to CSKp and CRKp infection were evaluated in this study. The mortality of *K. pneumoniae* BSI patients was associated with a higher APACHE II score, liver failure, and trachea cannula on the day of bacteremia, but not with carbapenem resistance. In another study, multivariate analyses revealed that carbapenem resistance was not a risk factor for mortality due to *K. pneumoniae* bacteremia [33]. The mortality rate of CRKp patients was significantly higher than that of CSKp patients in that study, which contradicts our findings and those of a study conducted in Israel in 2012 [46]. The risk factors for mortality due to *K. pneumoniae* BSI vary among studies, and include bedridden status, chronic liver disease, Charlson comorbidity index ≥5, mechanical ventilation, hemodialysis [33], and Pitt bacteremia score [46].

PFGE and MLST revealed that the isolates had considerable clonality; the 33 isolates were of different origins. No suspected outbreaks occurred during the study period. We assessed the frequency of 10 virulence genes in the CSKp and CRKp BSI isolates. *magA* is involved in the production of the K1 capsule, which is an important virulence factor [47]. A previous study confirmed local emergence of *K. pneumoniae* invasive syndrome and implicated *magA* and *rmpA* in its pathogenesis [48]. A number of putative virulence factors, including *magA* and *rmpA*, are associated with hypermucoviscous *K. pneumoniae* (hvKP), which can cause serious infections [49, 50]. Alarmingly, multidrug-resistant, including carbapenem-resistant hvKP isolates have emerged [23, 51, 52]; thus, we assessed the frequency rates of various virulence genes in the CRKp and CSKp groups. The frequency rates of *allS* and *magA* were higher in the CSKp group than in the CRKp group, despite the small number of subjects. Therefore, further studies of virulence genes, possibly using whole-genome sequencing (which is becoming less costly and more rapid) and involving larger populations, are warranted.

Our study had several limitations. First, relatively few patients were enrolled, which hampered the multivariate analysis and ability to draw firm conclusions. Second, some *K. pneumoniae* isolates were not stored. Third, the therapeutic regimen for *K. pneumoniae* BSI was not taken into consideration. Fourth, in the ICU setting, a heterogeneous population can limit the statistical analysis. Fifth, the role of the immune system was not analyzed. Despite these limitations, however, we identified several differences between the CRKp and CSKp groups, and explored the impact of carbapenem resistance and bacterial virulence genes on the outcomes of patients with *K. pneumoniae* BSIs.These data can lay the groundwork for future research in this field.

## Conclusions

An indwelling central venous catheter is a risk factor for CRKp BIS. Liver and kidney function at admission were lower in the CSKp group than in the CRKp group in this study. The mortality rate and frequency of bacterial virulence genes were similar between the CSKp and CRKp groups. Mortality due to *K. pneumoniae* BSI was not related to carbapenem resistance in univariate analysis. Further study is required to verify the correlation between CRKp-mortality mortality and the virulence genes of *K. pneumoniae* isolates.

## Abbreviations

ALT: Alanine transaminase
APACHE II: Acute Physiology and Chronic Health Evaluation
AST: Aspartate transaminase
BSIs: Bloodstream infections
CI: Confidence interval
CL: Central line
CLSI: Clinical and laboratory standards institute
CRE: Carbapenem-resistant Enterobacteriaceae
CRKp: Carbapenem-resistant *K. pneumoniae*
CRP: C-reactive protein
CSKp: Carbapenem-susceptible *K. pneumoniae*
HIV: Human immunodeficiency virus
HR: Hazard ratio
hvKP: Hypermucoviscous *K. pneumoniae*
ICU: Intensive care unit
MLST: Multilocus sequence typing
OR: Odds ratio
PCR: Polymerase chain reaction
PFGE: Pulsed-field gel electrophoresis
PT: Prothrombin time
SIRS: Systemic inflammatory response syndrome
SOFA: Sequential organ failure assessment
ST: Sequence typing
UTIs: Urinary tract infections
UTIs: Urinary tract infections
WBC: White blood cell
